# Treatment of osteoarthritis with autologous, micro-fragmented adipose tissue: a study protocol for a randomized controlled trial

**DOI:** 10.1186/s13063-021-05628-4

**Published:** 2021-10-27

**Authors:** Rasmus Kramer Mikkelsen, Lars Blønd, Lisbeth Rosenkrantz Hölmich, Cecilie Mølgaard, Anders Troelsen, Per Hölmich, Kristoffer Weisskirchner Barfod

**Affiliations:** 1 Department of Orthopaedic Surgery, Copenhagen University Hospital Hvidovre, Kettegård Allé 30, 2650 Hvidovre, Denmark; 2 Sports Orthopedic Research Center – Copenhagen (SORC-C), Hvidovre, Denmark; 3 Department of Orthopedic Surgery, University Hospital Zealand, Lykkebækvej 1, 4600 Køge, Denmark; 4 Department of Plastic Surgery, Copenhagen University Herlev and Gentofte Hospital, Borgmester Ib Juuls Vej 1, 2730 Herlev, Denmark; 5 Department of Clinical Medicine, University of Copenhagen, Copenhagen, Denmark; 6 Clinical Orthopedic Research Hvidovre (CORH), Hvidovre, Denmark

**Keywords:** Knee osteoarthritis, Stemcell, Pericyte, Biologic treatment, MSCs, Micro-fragmented adipose tissue, Randomized controlled trial

## Abstract

**Background:**

Osteoarthritis is a destructive joint disease that leads to degeneration of cartilage and other morphological changes in the joint. No medical treatment currently exists that can reverse these morphological changes. Intra-articular injection with autologous, micro-fragmented adipose tissue has been suggested to relieve symptoms.

**Methods/Design:**

The study is a blinded randomized controlled trial with patients allocated in a 1:1 ratio to 2 parallel groups. Patients suffering from pain and functional impairment due to osteoarthritis Kellgren-Lawrence grades 2–3 in the tibiofemoral joint are eligible for inclusion. The intervention group is treated with an intra-articular injection with autologous, micro-fragmented adipose tissue prepared using the Lipogems® system. The control group receives an intra-articular injection with isotonic saline. In total, 120 patients are to be included.

The primary outcome is The Knee injury and Osteoarthritis Outcome Score (KOOS4) evaluated at 6 months. Secondary outcomes are KOOS at 3, 12 and 24 months; the Tegner activity score; treatment failure; and work status of the patient. The analysis will be conducted both as intention-to-treat and per-protocol analysis.

**Discussion:**

This trial is the first to investigate the efficacy of autologous, micro-fragmented adipose tissue in a randomized controlled trial. The study uses the patient-reported outcome measure Knee Injury and Osteoarthritis Outcome Score (KOOS4) after 6 months as the primary outcome, as it is believed to be a valid measure to assess the patient’s opinion about their knee and associated problems when suffering from osteoarthritis.

## Administrative information

Note: the numbers in curly brackets in this protocol refer to SPIRIT checklist item numbers. The order of the items has been modified to group similar items (see http://www.equator-network.org/reporting-guidelines/spirit-2013-statement-defining-standard-protocol-items-for-clinical-trials/).

**Table:** 

Title {1}	Treatment of Osteoarthritis with Autologous, micro fragmented Adipose Tissue: A study protocol for a randomized controlled trial
Trial registration {2a and 2b}.	Trial registration ClinicalTrials.gov: NCT03771989 Registered on Dec. 13th 2018.
Protocol version {3}	Protocol version 1, Dec. 13^th^ 2018
Funding {4}	The study’s main sponsors are the department of Orthopedic Surgery at Copenhagen University Hospital Hvidovre and Zealand University Hospital. Lipogems® are delivering the equipment free of charge for the study.
Author details {5a}	Rasmus Kramer Mikkelsen: rasmus.kramer.mikkelsen.02@regionh.dk Lars Blønd: larbl@regionsjaelland.dk Lisbet Rosenkrantz Hölmich: lisbet.rosenkrantz.hoelmich@regionh.dk Cecilie Mølgaard: cekr1990@gmail.com Per Hölmich: per.hoelmich@regionh.dk Anders Troelsen: anders.troelsen@regionh.dk Kristoffer Weisskirchner Barfod: kristoffer.weisskirchner.barfod.02@regionh.dk ^1^Department of Orthopadic Surgery, Copenhagen University Hospital Hvidovre, Kettegård Allé 30, 2650 Hvidovre, Denmark. ^2^Department of Orthopedic Surgery, University Hospital Zealand, Lykkebækvej 1, 4600 Køge. ^3^Department of Plastic Surgery at Copenhagen University Herlev and Gentofte Hospital, Borgmester Ib Juuls Vej 1, 2730 Herlev. ^4^Sports Orthopedic Research Center – Copenhagen (SORC-C), ^5^Clinical Orthopedic Research Hvidovre (CORH). 6. Department of Clinical Medicine, University of Copenhagen, Copenhagen, Denmark Lipogems, Giovannino Barbieri
Name and contact information for the trial sponsor {5b}	Giovannino Barbieri Lipogems International Spa giovannino.barbieri@lipogems.eu Dept. Of Orthopaedic Surgery, Hvidovre Kettegård Allé 30, 2650 Hvidovre, Denmark
Role of sponsor {5c}	The role of Lipogems the company was to deliver the equipment for the study, which they are doing free of charge. The study’s main sponsors are the department of Orthopedic Surgery at Copenhagen University Hospital Hvidovre and Zealand. Their role were to help facilitate operating rooms for the study.

## Introduction

### Background and rationale {6a}

Osteoarthritis (OA) of the knee is a destructive joint disease, seen with increasing age, causing degeneration of cartilage, damage to the underlying bone and morphological changes to the joint [1]. It is a major public health concern due to the increased life expectancy of the ageing population [2, 3]. No approved medical treatment currently exists that reverses the morphological changes. Conventional treatment includes physiotherapy, pain medicine, braces and in end-stage OA surgical knee replacement [4].

During the past decade, researchers have started to explore the regenerative potential of mesenchymal stem cells (MSC) in OA [5]. MSCs are multipotent progenitor cells able to give rise to osteocytes, adipocytes, chondrocytes, myoblasts and tenocytes [6]. MSCs were first used to treat Chondral defects in 1998 [7] and to treat OA in 2002 [8]. Since then, a number of case reports and prospective series have been published showing significant short- and long-term effects on pain and cartilage thickness [5]. Recently, a prospective case-series of 1128 patients involving 1856 joints found an improvement of at least 50% in 86% of patients at 3 months and 91% of patients at 12 months using a modified Knee/Hip Osteoarthritis Outcome Score (KOOS/HOOS) questionnaire. Only 0.9% of patients did not show improvement after treatment [9]. However, when interpreting these studies, one should bear in mind that they include no controls and are level-four evidence and as such prone to bias.

No serious adverse effects like infection or neoplastic formation have been observed in treatment of OA with MSC [9, 10]. Non-manipulated or minimally manipulated cell therapies have been used within a wide range of medical conditions including stroke, myocardial infarction, Crohn’s disease, rheumatoid arthritis and breast augmentation. More than 17,000 scientific articles have been published reporting treatment of more than 320,000 patients [11]. No severe safety issues have been raised [12].

Mesenchymal stem cells can be derived from bone marrow or adipose tissue [5]. Most research has been performed on bone marrow-derived stem cells. Studies have shown promising results, but in 2016 a randomized study found no effect of active treatment with bone marrow aspirate concentrate compared to placebo with saline in the treatment of osteoarthritic knees [13]. A series of studies have recently been published showing promising effect of adipose-derived mesenchymal stem cells (AMSCs). By using AMSC, a large number of cells can be harvested from a small volume of tissue thereby avoiding the costly and time consuming process of expanding the cells in culture [5, 9].

Mesenchymal stem cells are thought to be able to activate and influence the microenvironment by serving as “a site-regulated drug store” [14]. Caplan and Correa use the term Medicinal Signaling cells (MSC), instead of mesenchymal stem cells, due to the in vivo qualities of those cells. The use of adipose-derived mesenchymal stem cells in treatment of OA has been of huge interest the past years [15, 16], and the complex regulatory issues involved in using enzymatic treated and/or expanded cells have led to the development of minimally manipulated tissue techniques [17]. The Lipogems system is one such system where the adipose tissue is micro-fragmented and washed free of blood residues. The resulting product is safe (FDA approval in 2016) and is said to be effective in the treatment of different pathologies [18], but level one evidence is lacking.

Initially, a pilot safety study was performed and no serious adverse effect was observed [19]. In this study, a positive result was found in 15 out of 20 patients. The pilot study was a prospective study, but not a randomized controlled study.

### Objectives {7}

With this study, we aim to investigate if treatment of patients with osteoarthritis of the knee with autologous, micro-fragmented adipose tissue prepared using the Lipogems system improves the patient reported health and function.

Hypothesis: Treatment of patients with osteoarthritis of the knee with autologous, micro-fragmented adipose tissue prepared using the Lipogems system leads to (1) improved patient-reported health seen as an increase in KOOS4 and (2) improved physical activity of the patient seen as an increase in the Tegner activity score.

Null hypothesis: There is no difference in patient-reported outcome or physical activity after treatment with autologous, micro-fragmented adipose tissue when compared to placebo treatment.

### Trial design {8}

The study is performed as a blinded, randomized controlled trial (RCT). Patients are individually randomized in two parallel groups in a 1:1 ratio:

1. The intervention group: Participants are treated with a 10-ml intra-articular injection of autologous, micro-fragmented adipose tissue.

## The control group: Participants receive a 10-ml intra-articular injection with isotonic saline (placebo).

## Methods: participants, interventions and outcomes

### Study setting {9}

The study will be performed at two centres in Denmark, Copenhagen University Hospital Hvidovre and Zealand University Hospital, as these two hospitals are the workplace of the authors.

### Eligibility criteria {10}

Patients with pain and functional impairment due to knee osteoarthritis are eligible for inclusion.

**Fig. 1:**
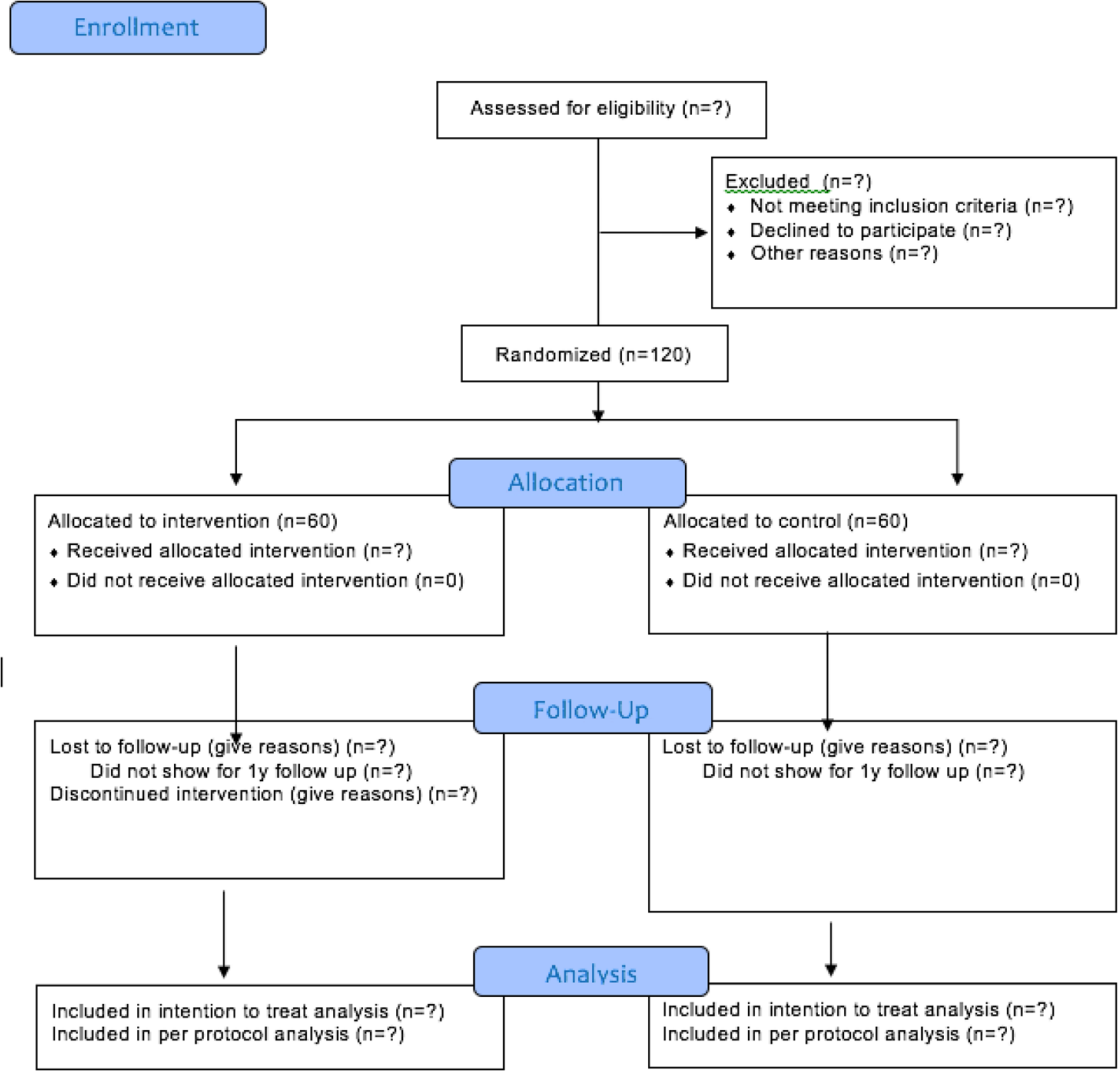
Patient flow diagram. Patients assessed for eligibility are registered according to the consort requirements and will be presented in the final study paper in a consort flow diagram like this

If the arthroplasty or sports surgeon, after clinical assessment, finds a patient eligible for inclusion, the patient is referred to the primary investigator for verbal information and written information concerning the trial. The patient is given the opportunity, on an informed basis, to decide whether he/she wants to participate in the trial and is informed of his/her right to at least 24 h of reflection before deciding. See Fig. 1 for patient flow diagram.

All patients assessed by the primary investigator for eligibility are registered according to the consort requirements.

If a patient meets the inclusion criteria, and has signed the declaration of consent, baseline registration is recorded by the treating surgeon in a REDcap database designed specifically for the study. After recording baseline data, a survey is automatically sent to the patient by email. The survey includes baseline KOOS4, Tegner activity score and questions regarding work status.

The inclusion criteria are as follows:

* Age 18–70 years.
* Kellgren-Lawrence score grades 2–3 in the tibiofemoral joint, either in the * medial, lateral or both compartments.
* The patient must be expected to be able to attend follow-up examinations.
* The patient must be able to speak and understand Danish.
* The patient must be able to give informed consent.

Exclusion criteria are:

* Smoking (the patient must consent to being non-smoking 6 weeks before and 6 weeks after the procedure)
* Varus or valgus malalignment of the knee > 5°
* Laxity of the medial collateral ligament (MCL) or lateral collateral ligament (LCL) of 10° or more compared to the opposite knee
* Knee instability and anterior-posterior laxity of 3 mm or above (compared to the opposite knee)
* Preceding open surgery to the knee or fracture in the proximal tibia or distal femur
* Extension deficit of the knee
* Kellgren-Lawrence score grade 4 in any of the three compartments
* BMI > 40
* Terminal illness or severe medical illness: ASA score higher than or equal to 3

### Who will take informed consent? {26a}

If the arthroplasty or sports surgeon, after clinical assessment, finds a patient eligible for inclusion, the patient is referred to the primary investigator for verbal information and written information concerning the trial. The patient is given the opportunity, on an informed basis, to decide whether he/she wants to participate in the trial and is informed of his/her right to at least 24 h of reflection before deciding. See Fig. 1 for patient flow diagram.

All patients are assessed by the primary investigator for eligibility.

### Additional consent provisions for collection and use of participant data and biological specimens {26b}

All patients were informed verbally and by written information, that 2 ml of the micro-fragmented tissue is stored in a freezer at a temperature of – 81 °C for cell count and later analysis of bioactive markers. By the end of the project, any biological material left will be destroyed.

### Interventions

#### Explanation for the choice of comparators {6b}

The intervention group: Participants are treated with a 10-ml intra-articular injection of autologous, micro-fragmented adipose tissue.

The control group: Participants receive a 10-ml intra-articular injection with isotonic saline (placebo).

The aim of the study is to find out if the active treatment is better than no treatment. A saline injection was chosen as the comparator as it was considered the best alternative to mimick “no treatment” in a blinded setup.

#### Intervention description {11a}

Description of the active treatment:

The active treatment is an intra-articular injection with autologous, micro-fragmented adipose tissue prepared using the Lipogems® system.

Harvest of adipose tissue is performed in local anaesthesia under sterile conditions in the operating theatre. The patient is positioned supine, and the lower abdomen is used as donor site, and this is marked on the skin with a surgical marker. The area is approximately 8 cm (crania-caudal) × 20 cm (lateral) and situated just below the umbilicus. In patients, where tissue harvest from the abdominal area is not possible due to low fat mass or scarring from earlier procedures, harvest is performed from the lateral thigh area. Surgical cleaning is performed twice and a sterile draping is applied. The adipose tissue is prepared for harvesting by injection of tumescence using a disposable 17G blunt cannula connected to a suspension of 40 ml Carbocain 1%, 250 ml isotonic saline, 0.5 mg adrenaline and 10mmol Bicarbonate. Fifteen minutes after installation of the local anaesthesia, approximately 100-ml fat is harvested manually using a 13G blunt suction cannula connected to a Vaclock® 20-ml syringe via two stab punctures. At the end of the procedure, the skin is closed with a band aid, and the patient is given an elastic compression bandage to be used for 3–4 weeks or as long as the area of liposuction is sore and swollen.

The harvested fat is immediately processed in the Lipogems® processing kit, a disposable closed device that progressively and mechanically reduces the size of the adipose tissue clusters while eliminating blood residues with pro-inflammatory properties during constant irrigation. The entire process, carried out in one surgical step, is performed in complete immersion in physiological saline solution minimizing traumatic action on the important cell products. The resulting micro-fragmented tissue is collected in two 5-ml syringes to be re-injected in the patient’s knee joint.

Implantation of the graft is performed with the patient supine using two injections sites in order to reduce the risk of extra-articular injection. Local anesthesia with lidocaine 1% is given in the skin at the two injection areas. The first injection is in the intercondylar notch. On a 90° bend knee the lateral soft spot is located just lateral to the patella tendon and approximately 1 cm proximal to the tibial plateau. The syringe is introduced 2–4 cm perpendicular to the tibial axis aiming in the direction of the cruciate ligaments. To make sure the needle is positioned intra-articular, 1 ml of saline is injected; if there is any resistance to the injection, the needle is repositioned and the procedure done over again. Injection is performed using a 21-gauge syringe. The second injection is done with the knee in full extension. The syringe is introduced from lateral into the suprapatellar pouch posterior to the patella. Injection of 1 ml saline is repeated to ensure intra-articular position. At the end of the procedure the skin is closed with two band aids.

#### Criteria for discontinuing or modifying allocated interventions {11b}

The trial is a one-time intervention. The intervention cannot be discontinued or modified.

#### Strategies to improve adherence to interventions {11c}

Not applicable since it’s a onetime intervention.

#### Relevant concomitant care permitted or prohibited during the trial {11d}

Participants are allowed standard pain medication and physiotherapy. Any type of intra-articular injection or operation is not permitted and will lead to exclusion from the trial.

#### Provisions for post-trial care {30}

There are no provisions for the participants in the trial.

Patients are covered by the patient insurance of Copenhagen University Hospital Køge and Hvidovre Hospital respectively.

### Outcomes {12}

#### Primary outcome

The primary outcome, the Knee injury and Osteoarthritis Outcome Score (KOOS_4_), is evaluated at 6 months after the intervention. See Fig. 3 for the study timeline.

The KOOS questionnaire was developed in the 1990s as an instrument to assess the opinion of patient’s with knee osteoarthritis about their knee and associated problems. Since the first publication in 1998, the psychometric properties of the KOOS have been assessed in more than twenty individual studies from all over the world. Furthermore, KOOS 1 year post surgery has been evaluated and compared to other instruments in several reviews [20–23].

KOOS4 constructs an average score for 4 out of the 5 KOOS subscale scores. It was first used by Frobell et al. in 2010 [24]. The fifth subscale concerning activities of daily living (ADL) is excluded in the KOOS 4 as the subscale is thought to add unwanted “noise” to the constructed outcome in active patients with few to none difficulties within ADL [23]. As the population of the trial is active patients, KOOS4 was chosen as the primary outcome.

#### Secondary outcomes (recorded at 3, 6, 12 and 24 months)

##### The Knee injury and Osteoarthritis Outcome Score (including all 5 subscales)

The KOOS holds 42 items in 5 separately scored subscales: KOOS Pain, KOOS Symptoms, Function in daily living (KOOS ADL), Function in Sport and Recreation (KOOS Sport/Rec) and knee-related Quality of Life (KOOS QOL) [23, 25].

The 5 patient-relevant subscales of KOOS are scored separately: KOOS Pain (9 items), KOOS Symptoms (7 items), KOOS ADL (17 items), KOOS Sport/Rec (5 items) and KOOS QOL (4 items). A Likert scale is used and all items have 5 possible answer options scored from 0 (No Problems) to 4 (Extreme Problems) and each of the 5 scores is calculated as the sum of the items included. Scores are transformed to a 0–100 scale, with 0 representing extreme knee problems and 100 representing no knee problems as is common in orthopaedic assessment scales and generic measures. Scores between 0 and 100 represent the percentage of total possible score achieved [23].

The Knee injury and Osteoarthritis Outcome Score is recorded at each follow-up.

##### Tegner Activity scale

The Tegner activity scale was described in 1985 [26] and was designed for ACL and meniscal injuries. The Tegner activity scale has been frequently used as a patient-administered activity rating for patients with different knee disorders. The Tegner score is a patient-administered score of activity level with 11 defined grades, from 0 representing disability because of knee injury to 10 (professional level soccer) [26].

Tegner activity score is recorded at each follow-up.

##### Work status

Changes in work status. Is the patient working full- or part time or not working. Our study groups are primarily patients who are eligible to work, and not patients already retired.

The patients are asked if they work full time, part time or not working. The patient answers if the work is hard physical work, moderate physical or light physical work.

Work status is recorded at each follow up.

##### Participation in sport and physical activity

Does the patient participate in sports or other forms of physical activity? Does the level of physical activity rise or fall after treatment? This is recorded at each follow-up. The patients are asked what type of physical activity they participate in, and how many hours a week.

##### Donor site morbidity

At 3 months it is recorded if an infection in the knee or at the donor site has occurred.

Recall VAS pain scale for the donor site first week after surgery, after 2 weeks, after 1 month and at present (3 months).

At 6 months, donor site morbidity is recorded as VAS pain scale for the donor site.

##### Failure

If the patient does not experience a clinically relevant improvement in KOOS_4_, the treatment is considered a failure, and this is registered at follow-up. A clinically relevant difference is estimated to be 10 points [24]. The number of patients experiencing failure of the treatment is recorded.

### Participant timeline {13}

**Fig. 2:**
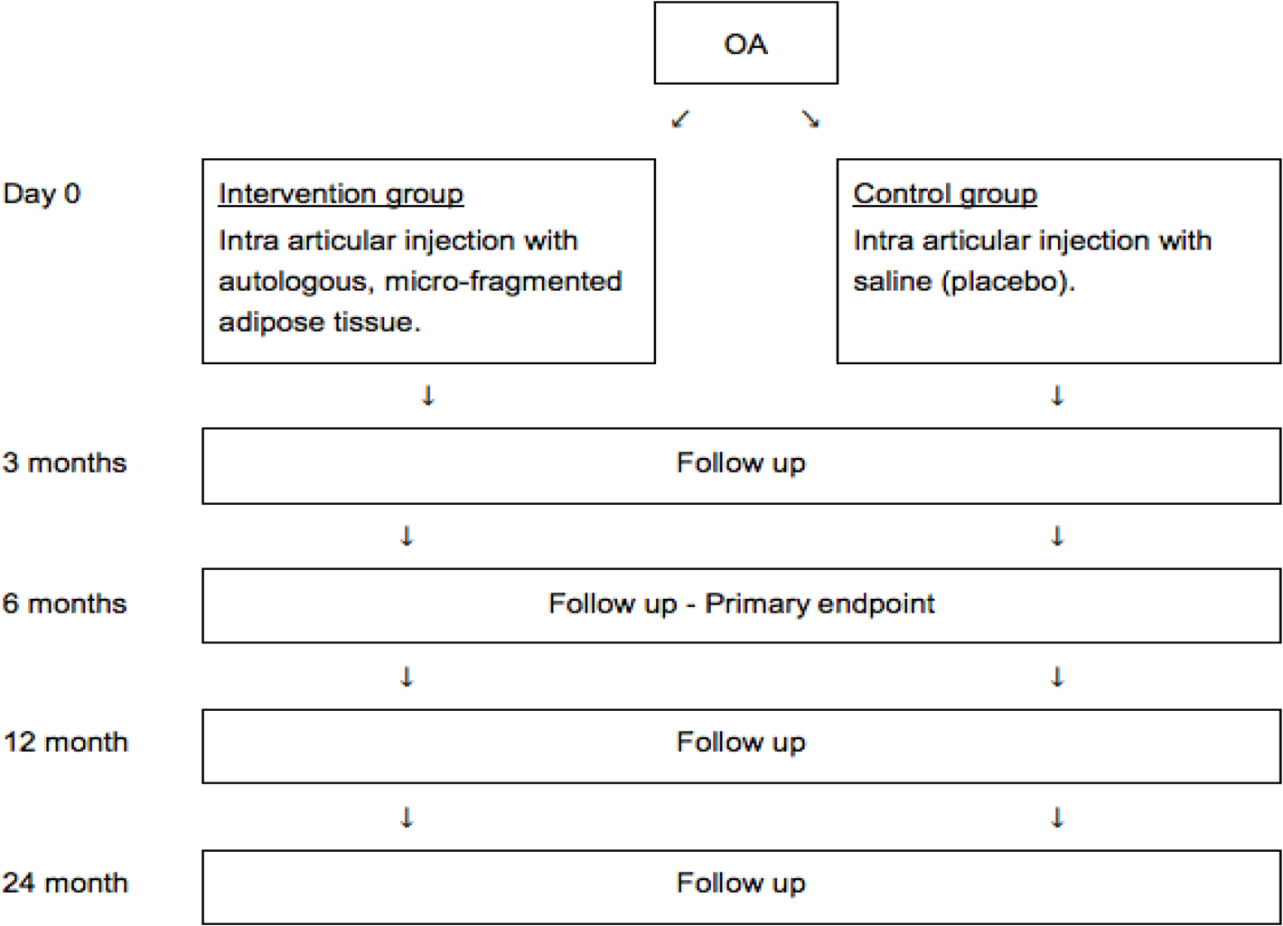
Participant time line showing the patient contacts in the study: randomization, intervention and follow-up

Participant timeline is shown in Fig. 2.

### Sample size {14}

One hundred twenty patients will be included, 60 patients in each group. The sample size calculation is based on a clinical relevant difference of 10 point in KOOS4, a standard deviation (SD) of 15 and power of 0.90 (two sided). Ninety-eight patients are required; due to the risk of dropout, 120 patients will be included, 60 in each group. The used SD and clinically relevant difference are recommended at KOOS.nu.

### Recruitment {15}

Participants are recruited from the arthroplasty and sports surgery departments of each hospital. Both departments have many patient contacts, which will give a continuous enrolment of possible participants.

## Assignment of interventions: allocation

### Sequence generation {16a}

Randomization is computer based, performed in blocks of twelve and is following the random allocation rule to ensure balanced group sizes. Patients are allocated 1:1 to either the control or the intervention group.

### Concealment mechanism {16b}

Two researchers with no other connection to the trial is responsible for packing of continuously numbered, sealed, opaque envelopes. The allocation key is stored by and only accessible by the researcher who generated the allocation key. If a participant needs to know the allocated treatment ahead of trial termination, the study nurse will arrange this. Patients are still followed up according to the intention to treat principle.

### Implementation {16c}

An experienced senior researcher with no other connection to the trial is responsible for generation of the allocation key.

## Assignment of interventions: blinding

### Who will be blinded {17a}

Blinding of the patient is secured as follows: The liposuction and micro-fragmentation of the fatty tissue is performed in the operating theatre. When the graft is ready for use, two 5-ml syringes containing active treatment and two 5-ml syringes containing saline are prepared.

The randomization envelope is opened by the treating surgeon, who is not blinded to the intervention.

The patient as well as the scrub nurse is blinded to the treatment. Both are visually shielded by the surgical drapes. Before opening the randomization envelope, the patient is informed that after opening of the envelope there is no further communication with the treating surgeon. The surgeon is silent once the randomization has taken place to limit subliminal bias. After the intra-articular injection of either micro-fragmented fat or saline, the surgeon empties the remaining syringes in order to conceal the given treatment.

Injection is performed with a 21G cannula. Due to the difference in viscosity of the graft and saline, the treating surgeon cannot be blinded.

In order to investigate if the blinding is working, the patients will be asked at 6 months’ follow-up which group he/she believes he/she was assigned to.

The data assessment is blinded as the data analysts are blinded.

### Procedure for unblinding if needed {17b}

The participants can ask to be unblinded upon request. The project manager will ask the person responsible for generation of the allocation key. The person has no other connection to the study.

## Data collection and management

### Plans for assessment and collection of outcomes {18a}

The Redcap database makes it easy to assess the correctness and completeness of the assembled data.

### Plans to promote participant retention and complete follow-up {18b}

The study nurse will be responsible for follow-up. The database will show if the participants have completed the questionnaires. If the questionnaire is not completed, the study nurse will make a phone call to the participant.

### Data management {19}

#### Data collection methods

Study data are collected and managed using REDCap electronic data capture tools hosted at the capital region of Denmark [27, 28].. REDCap (Research Electronic Data Capture) is a secure, web-based software platform designed to support data capture for research studies.

Baseline data about the patient is entered by the investigating surgeon. Data is entered directly in the database, ensuring secure storage of data and reducing the risks of typing errors, double data entry, data entry for a wrong patient, etc.

Information about the procedure including any complications is recorded by the treating surgeon in the REDcap database and level of pain during surgery is entered by the treating surgeon. The patient scores his/hers pain level on a visual analogue pain scale immediately after the surgery.

**Fig. 3:**
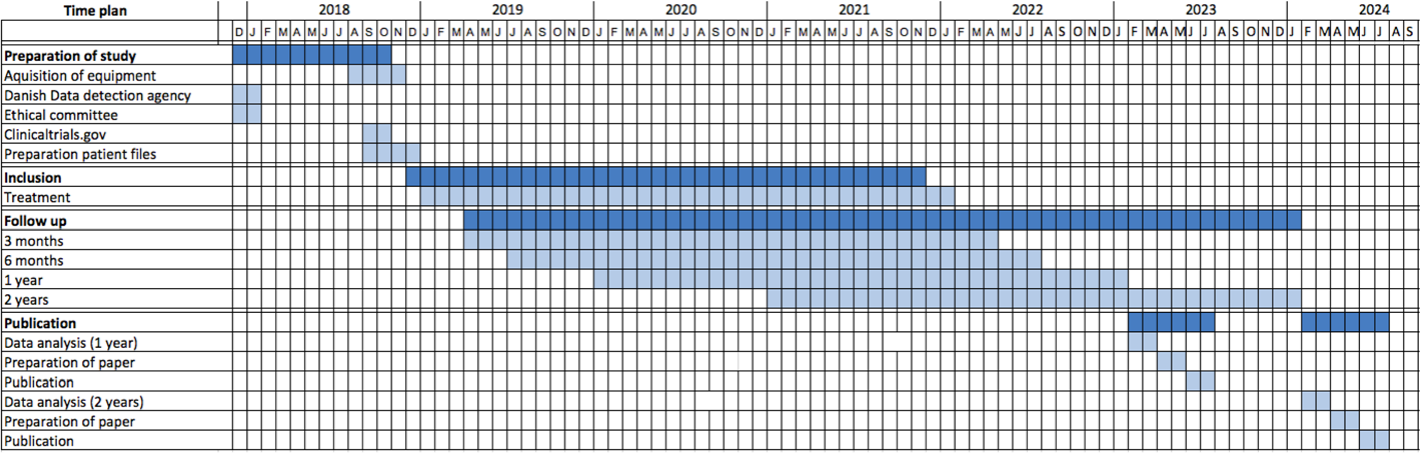
The study timeline. Inclusion of patients began in December 2018 and is expected to end November 2021

Data entry activates automated surveys to be sent to the patient at 3, 6, 12 and 24 months after the surgery. The patient will receive a link to the surveys by email and is contacted by phone by a study nurse to ensure completeness of the follow-up. The study nurse did not take part in the surgery and is blinded to the randomization. See Fig. 3 for the study timeline.

Collected data

**Table:** 

Data collection at:	Type of data	
**Baseline**	Patient-reported outcomes:	KOOS
		Tegner Activity score
	Work status	Working full time, part time or not working
		Type of work (hard, moderate or light physical labour)
	Sports participation	Participation in sport YES/NO
		Type of sport
		Number of hours per week.
	Patient demographic parameters	Name, social security number, age, gender, phone number, email
	Patient health data	ASA score, Diabetes, Hypertension, Rheumatic disease (RA or connective tissue disease), cardiovascular disease
	Knee-related data:	Kellgren Lawrence score
		Knee alignment
		Visual analogue scale for Pain (VAS pain) at rest and at hard physical activity.
**3 months**	Patient-reported outcomes	KOOS
		Tegner Activity score
	Work status	Working full time, part time or not working
		Type of work (hard, moderate or light physical labour)
	Sports participation	Participation in sport YES/NO
		Type of sport
		Number of hours per week.
	Registration of complications from the surgery and donor site morbidity	Infection in the knee or donor site
		Recall VAS pain scale for the donor site first week after surgery, after 2 weeks, after one month at present (3 months).
	Knee-related data	Visual analogue scale for Pain (VAS pain) at rest and at hard physical activity.
**6 months**	Patient-reported outcomes	KOOS
		Tegner Activity score
	Work status	Working full time, part time or not working
		Type of work (hard, moderate or light physical labour)
	Sports participation	Participation in sport YES/NO
		Type of sport
		Number of hours per week
	Knee-related data	Visual analogue scale for Pain (VAS pain) at rest and at hard physical activity.
	Donor site morbidity	VAS pain scale for the donor site
**12 and 24 months**	Patient-reported outcomes:	KOOS
		Tegner Activity score
	Work status	Working full time, part time or not working
		Type of work (hard, moderate or light physical labour)
	Sports participation	Participation in sport YES/NO
		Type of sport
		Number of hours per week
	Knee-related data:	Visual analogue scale for Pain (VAS pain) at rest and at hard physical activity.

### Confidentiality {27}

All information about patients is confidential, and information will only be shared between the people involved in the study.

Personal data is registered in the RedCap database where it is stored safely to protect confidentiality before, during and after the trial.

### Plans for collection, laboratory evaluation and storage of biological specimens for genetic or molecular analysis in this trial/future use {33}

Two millilitres of the micro-fragmented tissue is stored in a freezer at a temperature of −81 °C for cell count and analysis of bioactive markers. Collection is meant for future use in ancillary studies. By the end of the project, any biological material left will be destroyed.

## Statistical methods

### Statistical methods for primary and secondary outcomes {20a}

Demographic parameters and outcomes at baseline are presented descriptively for the two groups. Between-group comparison of the primary and secondary outcomes is performed by use of relevant statistics according to the characteristics and distribution of the variables. The primary outcome, KOOS, is continuous and expected to be normally distributed. Due to the study design with repeated measurements at baseline, 3, 6 and 12 months, analysis will be performed using a linear mixed effect model.

All statistical testing will be performed at the two-sided 5% significance level, and 95% confidence intervals will be presented where appropriate. Statistical testing will take place after all participants have completed their 1year follow-up and sufficient time has been allowed for data entry and validation.

### Interim analyses {21b}

No interim analyses are planned, and hence, no statistical testing will take place until the 1-year analysis.

### Methods for additional analyses (e.g. subgroup analyses) {20b}

No subgroup analyses are planned.

### Methods in analysis to handle protocol non-adherence and any statistical methods to handle missing data {20c}

Prior to any analysis, missing data pattern will be investigated and reasons for missing data obtained and summarized where possible. The primary analysis will be conducted as an intention-to-treat analysis, which includes all participants with missing outcome data, unless there is clear evidence that its underlying assumption is inappropriate. Sensitivity analysis will be performed to assess the robustness of the results by imputing missing data using multiple imputations under both missing at random and missing not at random assumptions. Per protocol analyses will also be performed.

### Plans to give access to the full protocol, participant-level data and statistical code {31c}

After the study is concluded, there are plans to make the anonymized dataset and statistical code publicly available.

## Oversight and monitoring

### Composition of the coordinating centre and trial steering committee {5d}

The study does not have a coordinating centre. The steering committee consist Lars Blond and Kristoffer W. Barfod.

### Composition of the data monitoring committee, its role and reporting structure {21a}

The study does not have a data monitoring committee.

The project will be reported to the Danish Data Protection Agency. The project will follow the Danish act concerning storage and handling of personal data.

The National Committee on Health Research Ethics and the Danish Health and Medicine Authority are allowed direct access to source data and documents (including medical records) when performing monitoring, auditing and / or inspection.

### Adverse event reporting and harms {22}

Follow-up is done by the study nurse. Harms such as infection and donor site morbidity are noted and reported to the project manager. Any adverse events or side-effect is reported immediately to the project manager.

### Frequency and plans for auditing trial conduct {23}

Auditing is not scheduled beforehand but The Danish Data Protection Agency can at all time schedule auditing.

### Plans for communicating important protocol amendments to relevant parties (e.g. trial participants, ethical committees) {25}

There are no plans to make amendments to the protocol. If that happens, both the ethics committee and participants will be informed by a written document.

## Dissemination plans {31a}

KWB will prepare the manuscript and be the first author. The other authors will appear as co-authors, if they at the time of submission fulfil the Vancouver rules for authorship.

The study will be sought published in an international, high-impact journal and presented at both national (Danish) and international medical conferences. There is public access to the full protocol, full anonymized dataset and statistical code.

The results will also be published online and in other relevant media. There are no publication restrictions.

## Trial status

This report is based on the second version of the study protocol dated the 15th of October 2018.

## Abbreviations

ACL: Anterior cruciate ligament
ADL: Activities of daily living
AMSCs: Adipose-derived mesenchymal stem cells
ASA: American Society of Anesthesiologists
BMI: Body mass index
CONSORT: Consolidated Standards of Reporting Trials
KOOS: The Knee injury and Osteoarthritis Outcome Score
LCL: Lateral collateral ligament
MCL: Medial collateral ligament
MSC: Mesenchymal stem cells
OA: Osteoarthritis
QOL: Quality of Life
RA: Rheumatoid arthritis
RCT: Randomized controlled trial
SD: Standard deviation
SPIRIT: Standard Protocol Items: Recommendations for Interventional Trials
VAS: Visual analogue scale
